# Performance Evaluation of Deep Learning Applications in Calcaneal Fracture Diagnosis Using Separate Radiographic Views: A Comparison of CNN and Vision Transformer Architectures

**DOI:** 10.3390/diagnostics16213682

**Published:** 2026-11-07

**Authors:** Shun-Ping Wang, Yu-Wei Chan, Yi-Cyuan Tseng, Wei-En Hsu

**Affiliations:** 1 Department of Orthopaedics, Taichung Veterans General Hospital, Taichung 407219, Taiwan; 2 Department of Post-Baccalaureate Medicine, College of Medicine, National Chung Hsing University, Taichung 40227, Taiwan; 3 Bachelor Degree Program of Artificial Intelligence, National Taichung University of Science and Technology, Taichung 404336, Taiwan; 4 Department of Electrical Engineering, National Chung Hsing University, Taichung 40227, Taiwan; 5 Department of Materials Science and Engineering, Feng Chia University, Taichung 407102, Taiwan

**Keywords:** calcaneal fracture, deep learning, convolutional neural networks, vision transformer, multi-view radiographs, explainable AI, Grad-CAM, computer-aided diagnosis

## Abstract

**Background:** Calcaneal fractures are challenging to diagnose on routine radiographs and may result in missed diagnoses. **Objective:** This study compares Convolutional Neural Networks (CNNs) and Vision Transformer (ViT) architectures for detecting calcaneal fractures separately in three standard radiographic views: Anteroposterior (AP), Harris (axial), and Lateral. **Methods:** A balanced retrospective dataset of 178 patients with complete three-view examinations (89 with displaced calcaneal fractures and 89 controls) was used to evaluate five deep learning models: ResNet50, InceptionV3, InceptionResNetV2, XCiT, and PiT. After image-quality screening, each view underwent an approximately 8:2 image-level training–validation split; no independent test set was used. Images were preprocessed using data augmentation and Contrast Limited Adaptive Histogram Equalization (CLAHE), while Gradient-weighted Class Activation Mapping (Grad-CAM) was employed to visualize model activation. **Results:** InceptionV3 achieved 100% sensitivity in the AP view, while ResNet50 achieved 100% specificity in both AP and Lateral views. XCiT matched ResNet50 across all five classification metrics in the AP view and matched ResNet50 and InceptionV3 in the Lateral view. PiT achieved 100% accuracy in the Harris validation set (95% CI, 89.0–100.0%). Grad-CAM visualizations showed activation over the calcaneal region in representative fracture images. **Conclusions:** This study demonstrates the feasibility of deep learning for identifying displaced calcaneal fractures through separate evaluations of AP, Harris, and Lateral radiographs. The model-specific performance patterns provide a foundation for further development of AI-assisted calcaneal fracture detection and future investigation of multi-view integration.

## 1. Introduction

Calcaneal fractures are the most common type of tarsal bone fractures, accounting for approximately 60% of all tarsal fractures and 2% of all systemic fractures [1,2,3]. Since improper management of calcaneal fractures can lead to long-term functional impairment, walking difficulties, and related complications, early and accurate diagnosis is crucial for patient prognosis. Although computed tomography (CT) provides precise diagnostic information and is widely used in the Sanders classification system to assess the severity of intra-articular fractures [3], radiography (X-ray) remains the primary tool for initial clinical evaluation. Routinely, three different projection views are utilized clinically—Anteroposterior (AP), Lateral, and Harris (Axial) views—to comprehensively assess the presence and severity of the fracture, further guiding subsequent examination and treatment strategies.

Despite the indispensable role of X-ray imaging in the diagnosis of calcaneal fractures, interpretation remains challenging for frontline clinicians. The literature indicates that the missed diagnosis rate of calcaneal fractures during initial preliminary diagnosis ranges between 3.7% and 10%, depending on the clinical scenario [4]. This is often attributed to indistinct fracture lines on X-ray images or the physician’s attention being diverted by other severe concomitant traumas (e.g., spinal compression fractures), known as distracting injuries [5]. Furthermore, studies have shown that the sensitivity of conventional X-rays for undisplaced or subtle fractures is approximately 87% [6], implying that a subset of cases may still be missed during initial examinations. Such missed diagnoses not only delay the optimal window for treatment but can also lead to severe complications such as malunion, deformity, and post-traumatic arthritis, potentially triggering medico-legal disputes and imposing dual burdens on both the patient and the healthcare system [2].

In recent years, the rapid development of artificial intelligence (AI) technology has brought new opportunities to medical image analysis [7], particularly Convolutional Neural Networks (CNNs) in the field of Deep Learning (DL) [8]. CNNs are currently the mainstream architecture for image processing, capable of extracting local image features through convolutional layers and compressing data via pooling layers to reduce computational costs, making them especially suitable for lesion detection and classification tasks. Transfer learning utilizing large datasets such as ImageNet has become a standard strategy to enhance the diagnostic performance of medical imaging [9]. Although studies have demonstrated the high efficacy of CNNs in the radiographic diagnosis of wrist [10], hip [11], and ankle fractures [12], or in calcaneal fracture diagnosis using X-ray images [13], research on AI applications specifically for the radiographic diagnosis of calcaneal fractures remains exceedingly scarce. Furthermore, the application potential of the emerging Vision Transformer (ViT) architecture in the medical imaging domain warrants exploration [14].

Given the aforementioned clinical diagnostic challenges and the gaps in existing literature regarding AI applications in calcaneal fractures—especially the lack of systematic comparisons concerning the adaptability of different deep learning architectures (CNN vs. Transformer) to the imaging characteristics of routine three-view radiographs—this study examines whether individual models exhibit different performance patterns across radiographic views: Considering that CNNs excel at extracting local detailed features, while Vision Transformer (ViT) architectures use attention-based feature modeling, we explore the performance of these architectures on X-ray views with distinct anatomical characteristics. Based on this objective this study aims to bridge the gap in the current literature by evaluating and comparing the diagnostic accuracy of five distinct deep learning models separately within three routine X-ray views of calcaneal fractures. Additionally, to address clinicians’ concerns regarding the opacity of AI decision-making processes (the “black-box” effect), this study introduces Grad-CAM (Gradient-weighted Class Activation Mapping) technology [15] to generate heatmaps. By visualizing model activation in representative fracture images, we aim to provide qualitative information about the spatial distribution of model activation.

## 2. Background Review and Related Work

### 2.1. Current State of AI in Fracture Diagnosis and the Calcaneal Research Gap

In recent years, AI, particularly DL models based on CNNs, has demonstrated remarkable success in the field of musculoskeletal radiology. Numerous studies have shown that AI can achieve diagnostic performance comparable to, or even exceeding, that of experienced radiologists in detecting common fractures such as those of the wrist, hip, and ankle [10,11,12,16]. These systems leverage large-scale datasets to learn complex pathological patterns, effectively reducing the workload of clinicians and minimizing the rate of missed diagnoses in emergency settings [4,17].

However, despite these broad advancements, a significant research gap remains concerning the radiographic diagnosis of calcaneal fractures. The calcaneus is a three-dimensionally complex structure, and its fractures often involve subtle cortical disruptions or joint surface depressions that are easily obscured by overlapping tarsal bones on two-dimensional X-rays. While current AI research has explored various anatomical sites, the literature specifically addressing calcaneal fractures is disproportionately sparse. Yu et al. [13] investigated multi-task learning for calcaneal fracture diagnosis using X-ray images, whereas Kuo et al. [16] provided a broader systematic review and meta-analysis of AI-based fracture detection.

The primary question in the current research landscape is how individual deep learning models perform across different radiographic projections. In clinical practice, a definitive diagnosis relies on the synergy of AP, Lateral, and Harris views, yet evaluating these projections separately and integrating them into a unified model are distinct research approaches. While Vision Transformer (ViT) architectures have emerged as a powerful alternative to CNNs using attention-based feature modeling—their specific efficacy and adaptability to the varying anatomical characteristics of different radiographic views (e.g., the global deformation in Harris views vs. local edges in Lateral views) warrant further investigation in the context of calcaneal fractures.

This study examines this question by evaluating five distinct deep learning architectures separately on AP, Harris, and Lateral radiographs. By comparing traditional CNNs with emerging Transformer-based models (XCiT and PiT), we aim to describe model-specific performance within each view under shared principal training conditions. Furthermore, this research reports results separately for each view and utilizes Grad-CAM [15] to visualize model activation in representative fracture images.

### 2.2. Deep Learning Model Architectures

This study selected five representative deep learning models for comparative analysis, encompassing classic CNN architectures and novel Vision Transformer technologies, to explore the applicability of different feature extraction mechanisms in fracture diagnosis:

ResNet50: Residual Neural Network (ResNet) introduces “Skip Connections” and Residual Block structures, allowing original input information to be passed directly to subsequent layers [8]. This design effectively resolves the vanishing gradient problem common in deep network training, making it possible to construct networks with over 50 layers. In this study, ResNet50 utilizes its deep architecture to extract high-level abstract features while retaining low-level details, which is crucial for identifying subtle structural features like fracture lines.

InceptionV3: InceptionV3 aims to resolve the contradiction between computational resources and model depth in image recognition. Its core is the Inception module, which simultaneously captures image features at different scales by using multiple convolutional kernels of varying sizes (e.g., 1 × 1, 3 × 3, 5 × 5) in parallel [18]. Moreover, InceptionV3 employs convolution factorization techniques, breaking down larger convolutional kernels (e.g., 7 × 7) into two one-dimensional convolutions (1 × 7 and 7 × 1), which dramatically reduces the number of parameters while increasing the non-linearity and depth of the network, making it particularly suitable for high-performance feature extraction in resource-constrained environments.

InceptionResNetV2: This model is a hybrid of the Inception architecture and the ResNet residual structure [19]. It inherits the multi-scale feature extraction capabilities of the Inception module while introducing ResNet’s residual connections to accelerate convergence and stabilize deep network training. For X-ray images, this hybrid architecture can theoretically capture both local bone texture details (via Inception) and global skeletal structural information (via deep residual networks) simultaneously, providing a more comprehensive feature representation for highly heterogeneous fracture images.

XCiT (Cross-Covariance Image Transformer): Unlike traditional Vision Transformers (ViT) that perform attention computations between image tokens (which leads to computational loads increasing quadratically with image resolution), XCiT proposes an innovative “Cross-Covariance Attention (XCA)” mechanism [20]. XCA operates across feature channels instead, resulting in a computational complexity that scales linearly with image resolution, making it more suitable for high-resolution medical images. Additionally, XCiT enhances the model’s ability to identify potential relationships between anatomical structures by calculating the cross-covariance matrix between features to capture global dependencies.

PiT (Pooling-based Vision Transformer): PiT is a Vision Transformer optimized for imaging tasks, designed to combine the strengths of both CNNs and Transformers [21]. Traditional ViTs maintain a fixed spatial resolution across all layers, ignoring the hierarchical nature of spatial structures. PiT introduces CNN-like pooling layers to progressively reduce spatial dimensions and increase channel counts at different network stages, forming a feature pyramid structure. This design progressively reduces spatial dimensions and builds hierarchical feature representations, enabling the model to capture image information at different spatial scales.

## 3. Materials and Methods

This study employed a retrospective deep learning framework to systematically compare the diagnostic performance of CNN- and Transformer-based architectures for calcaneal fracture detection using separate evaluations of three radiographic views. The methodological framework comprised patient cohort selection, radiographic image collection and preprocessing, dataset partitioning, model development and training, performance evaluation, and model interpretability analysis. Three standard radiographic views, including Anteroposterior (AP), Harris, and Lateral views, were independently analyzed to characterize model performance within each view.

**Figure 1:**
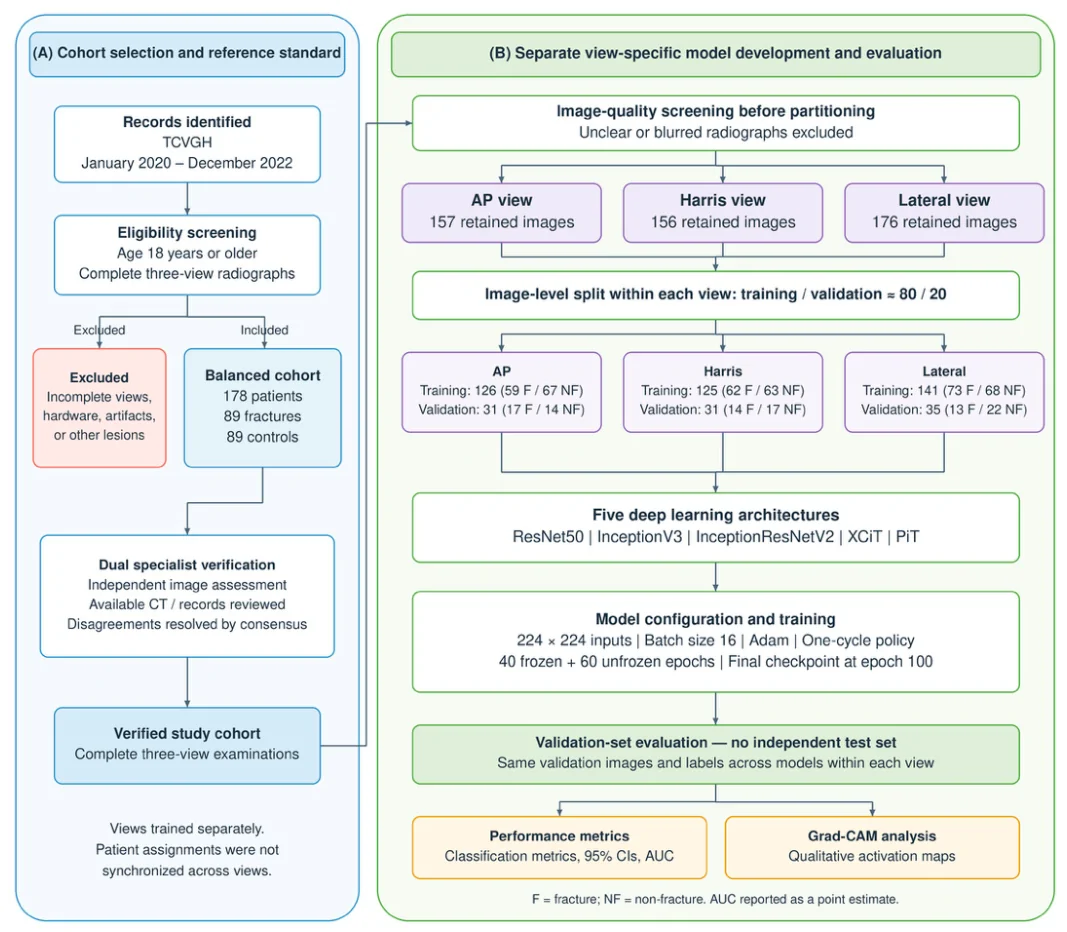
The overall study workflow.

Figure 1 presents an overview of the study workflow. Following cohort selection and establishment of the reference standard, individual radiographs underwent image-quality screening before the dataset was divided into training and validation sets using an approximately 8:2 image-level split within each view. Images from the three radiographic views were processed separately, before five deep learning architectures were trained. The trained models were evaluated on the validation set using standard performance metrics, and model interpretability was assessed using Grad-CAM. No independent test set or integrated multi-view model was evaluated. Detailed procedures are described in the following sections.

### 3.1. Image Collection and Dataset Construction

This study adopted a retrospective case–control study design, collecting X-ray imaging data from patients in a tertiary referral center between January 2020 and December 2022. Inclusion criteria were as follows: (1) age ≥ 18 years; (2) confirmed calcaneal fracture via X-ray or CT imaging; and (3) possession of a complete set of three-view X-ray images (Lateral, Harris, AP). Exclusion criteria included: (1) presence of internal fixation hardware in the ankle bones due to previous surgeries; (2) lack of complete three-view images; (3) severe artifacts in X-ray images interfering with interpretation; and (4) concomitant presence of other non-fracture bone lesions (e.g., bone tumors, cysts). All included fracture cases had displaced fractures.

To establish a balanced dataset, calcaneal fracture patients were selected based on the criteria, and an equal number of patients without radiographic evidence of calcaneal fracture were randomly selected as the control group. The original cohort comprised 89 fracture cases and 89 controls. Controls underwent radiographic examinations for other clinical indications and were not specifically recruited from patients presenting with acute heel or hindfoot trauma. Fracture exclusion in controls was based on plain radiographs alone.

Two senior orthopedic trauma specialists independently assessed the images to establish the reference labels. Available CT examinations and medical records were consulted when assessing fracture cases. Disagreements were resolved through joint discussion and re-examination of CT images when available, with the final reference label determined by consensus.

The analysis dataset contained radiographic images and fracture labels but did not include patient-level demographic and clinical variables for baseline characterization. Intra-articular versus extra-articular involvement and Sanders or Essex-Lopresti classifications were not systematically recorded. CT was available for some fracture cases, but the number confirmed by CT versus radiography alone was not recorded in the analysis dataset.

### 3.2. Dataset Partitioning and Image Preprocessing

All image files in this study were saved in JPG format and categorized according to diagnostic class (fracture/no fracture) and radiographic view (Lateral, Harris, AP). Before partitioning, individual radiographs were manually screened to exclude unclear or blurred images. The retained datasets comprised 157 AP images, 156 Harris images, and 176 Lateral images. Each view was randomly divided into training and validation sets using an approximately 8:2 image-level split. No independent test set was used.

For the AP view, the training set contained 126 images (59 fractures and 67 controls), and the validation set contained 31 images (17 fractures and 14 controls). For the Harris view, the training set contained 125 images (62 fractures and 63 controls), and the validation set contained 31 images (14 fractures and 17 controls). For the Lateral view, the training set contained 141 images (73 fractures and 68 controls), and the validation set contained 35 images (13 fractures and 22 controls). All reported performance metrics were obtained from these validation sets.

Each patient contributed one original image per view, and models were trained separately for each view. At the original-image level, assigning each image to a single subset therefore separated the contributing patients within each view. Patient assignments were not synchronized across views, so the three validation sets were not patient-matched. Within each view, all five architectures were evaluated using the same validation images and labels.

Data augmentation was implemented using fastai aug_transforms() [22]. The augmentation configuration included random rotation from −10° to +10° with an application probability of 0.75, horizontal flipping, random zoom from 1.0 to 1.1, warping, and brightness and contrast adjustments. No separate translation augmentation was applied. All models received three-channel images of 224 × 224 pixels. Images were processed using fastai Resize(224) in crop mode, with random crop positions during training and center cropping during validation. Furthermore, addressing the common issues of uneven exposure and low contrast in X-ray images, this study employed Contrast Limited Adaptive Histogram Equalization (CLAHE) technology [23]. Compared to traditional global histogram equalization, which tends to over-amplify background noise, CLAHE is designed to enhance the visibility of local details by performing equalization on local image patches and limiting contrast gain. Finally, normalization was applied using the fixed parameters associated with the corresponding pretrained configuration.

The reported results were obtained from a single random training–validation split for each view. Stratified cross-validation and repeated experiments with different random seeds were not performed.

### 3.3. Hardware, Software Configuration and Model Training

Model training and validation in this study were conducted in a computing environment. The hardware configuration utilized an Intel^®^ Core™ i7-8700 CPU paired with 32.0 GiB of RAM. The software environment was based on Python 3.8, utilizing PyTorch 2.0.1 and Fastai 2.7.12 as the deep learning frameworks.

ResNet50 was implemented using torchvision.models.resnet50 with ResNet50_Weights.DEFAULT (IMAGENET1K_V2). The remaining models were implemented using timm. InceptionV3 and InceptionResNetV2 used the inception_v3 and inception_resnet_v2 variants, with the pretrained configurations inception_v3.tv_in1k and inception_resnet_v2.tf_in1k, respectively. PiT used the pit_s_224 variant with pit_s_224.in1k pretrained weights, and XCiT used xcit_small_12_p16_224 with xcit_small_12_p16_224.fb_in1k pretrained weights. All models were initialized with ImageNet-1k pretrained weights.

Although the pretrained configurations of InceptionV3 and InceptionResNetV2 specify a reference input size of 299 × 299 pixels, both models were fine-tuned and evaluated using 224 × 224 inputs without additional resizing to 299 × 299. The pretrained timm convolutional backbones were retained, and spatial features were extracted using forward_features(). For 224 × 224 inputs, InceptionV3 and InceptionResNetV2 produced feature maps of 2048 × 5 × 5 and 1536 × 5 × 5, respectively. These features were passed to fastai binary classification heads. AdaptiveConcatPool2d concatenated the outputs of adaptive max pooling and adaptive average pooling, each with a spatial size of 1 × 1. After flattening, the classification heads had linear-layer dimensions of 4096 → 512 → 2 for InceptionV3 and 3072 → 512 → 2 for InceptionResNetV2. This configuration allowed the pretrained convolutional backbones to accommodate 224 × 224 inputs.

All models used a batch size of 16 and the fastai implementation of the Adam optimizer [24]. Training comprised two stages totaling 100 epochs. In the first stage, the pretrained backbone was frozen for 40 epochs, except for normalization parameters kept trainable under fastai’s train_bn=True setting. In the second stage, the entire network was unfrozen and fine-tuned for 60 epochs.

A one-cycle learning-rate policy was applied separately in each stage. The first stage used a maximum learning rate of 1 × 10^−3^. The second stage used discriminative maximum learning rates across parameter groups, specified as slice(1 × 10^−4^, 1 × 10^−3^) in fastai. No early stopping was applied; all models completed the fixed 100-epoch training schedule. All reported evaluations used the final checkpoint after 100 epochs, rather than the checkpoint with the lowest validation loss.

The principal training and optimization settings were shared across all five architectures and all three radiographic views.

### 3.4. Performance Evaluation Metrics

This study employed multiple classification performance metrics to evaluate the performance of the models in diagnosing calcaneal fractures. The evaluation was based on the confusion matrix, which includes four possible classification outcomes:

• True Positive (TP): Actual fracture correctly predicted as fracture.
• True Negative (TN): Actual no-fracture correctly predicted as no-fracture.
• False Positive (FP): Actual no-fracture incorrectly predicted as fracture.
• False Negative (FN): Actual fracture incorrectly predicted as no-fracture.

Based on the above definitions, this study calculated five key performance metrics using the following formulas:

Accuracy = (TP + TN)/(TP + TN + FP + FN)

Sensitivity (Recall) = TP/(TP + FN)

Specificity = TN/(TN + FP)

Precision = TP/(TP + FP)

F1-Score = 2 × (Precision × Sensitivity)/(Precision + Sensitivity)

Precision represents the positive predictive value within the evaluated validation sample. Receiver operating characteristic (ROC) curves and the area under the ROC curve (AUC) were also used to summarize discrimination across classification thresholds. Performance metrics were reported separately for each model and radiographic view.

### 3.5. Statistical Analysis

Performance estimates were reported with nominal 95% confidence intervals (CIs). CIs for sensitivity, specificity, precision, and accuracy were calculated from the corresponding confusion-matrix counts using the Wilson score method. CIs for the F1-score were calculated using the Wilson indirect method. Specifically, a Wilson score interval was first calculated for TP/(TP + FP + FN), and its lower and upper bounds were transformed using 2p/(1 + p) to obtain the F1-score interval.

These intervals describe image-level sampling uncertainty within the evaluated validation sets and do not account for variability arising from alternative dataset partitions or repeated model training. AUC values were reported as point estimates; AUC confidence intervals were not calculated.

Comparisons between models were descriptive. No paired hypothesis tests, such as McNemar’s test or DeLong’s test, were performed, and differences in point estimates were not interpreted as evidence of statistical superiority. Comparisons across radiographic views were also descriptive because the validation sets were not patient-matched.

### 3.6. Grad-CAM Analysis

Grad-CAM [15] was used to visualize model activation in representative fracture images. Heatmaps were generated from the activations and corresponding gradients at a specified target layer in each trained model. The target layers, identified by their paths in the saved models, were 0.7.2.conv3 for ResNet50, 0.model.Mixed_7c for InceptionV3, 0.model.conv2d_7b for InceptionResNetV2, 0.model.blocks.11.norm1 for XCiT, and 0.model.transformers.2.blocks.3.norm1 for PiT.

For XCiT, the target layer preceded class-token insertion and the class-attention stage. All 196 patch tokens were retained, and the activations and corresponding gradients were reshaped into tensors with 384 channels and spatial dimensions of 14 × 14. For PiT, the first token, corresponding to the class token, was removed. The remaining 49 patch tokens and their gradients were reshaped into tensors with 576 channels and spatial dimensions of 7 × 7.

For all architectures and examples, the pre-softmax fracture-class logit was used as the target score. Gradients were averaged over the spatial dimensions to obtain channel weights. A weighted sum of the activation maps was then calculated, followed by application of the rectified linear unit (ReLU). The resulting heatmaps were upsampled to 224 × 224 pixels.

The visualizations were examined qualitatively, with one representative fracture image presented for each radiographic view.

## 4. Results

Based on the inclusion and exclusion criteria, this study analyzed a total of 178 patients (89 in the fracture group and 89 in the control group), each with complete three-view examinations before image-quality screening. After screening, 157 AP, 156 Harris, and 176 Lateral images were retained. The corresponding validation sets contained 31, 31, and 35 images, respectively. The diagnostic performance of the five models (ResNet50, InceptionV3, InceptionResNetV2, XCiT, PiT) across different views is detailed below.

### 4.1. Diagnostic Performance of Models in the AP View

**Figure 2:**
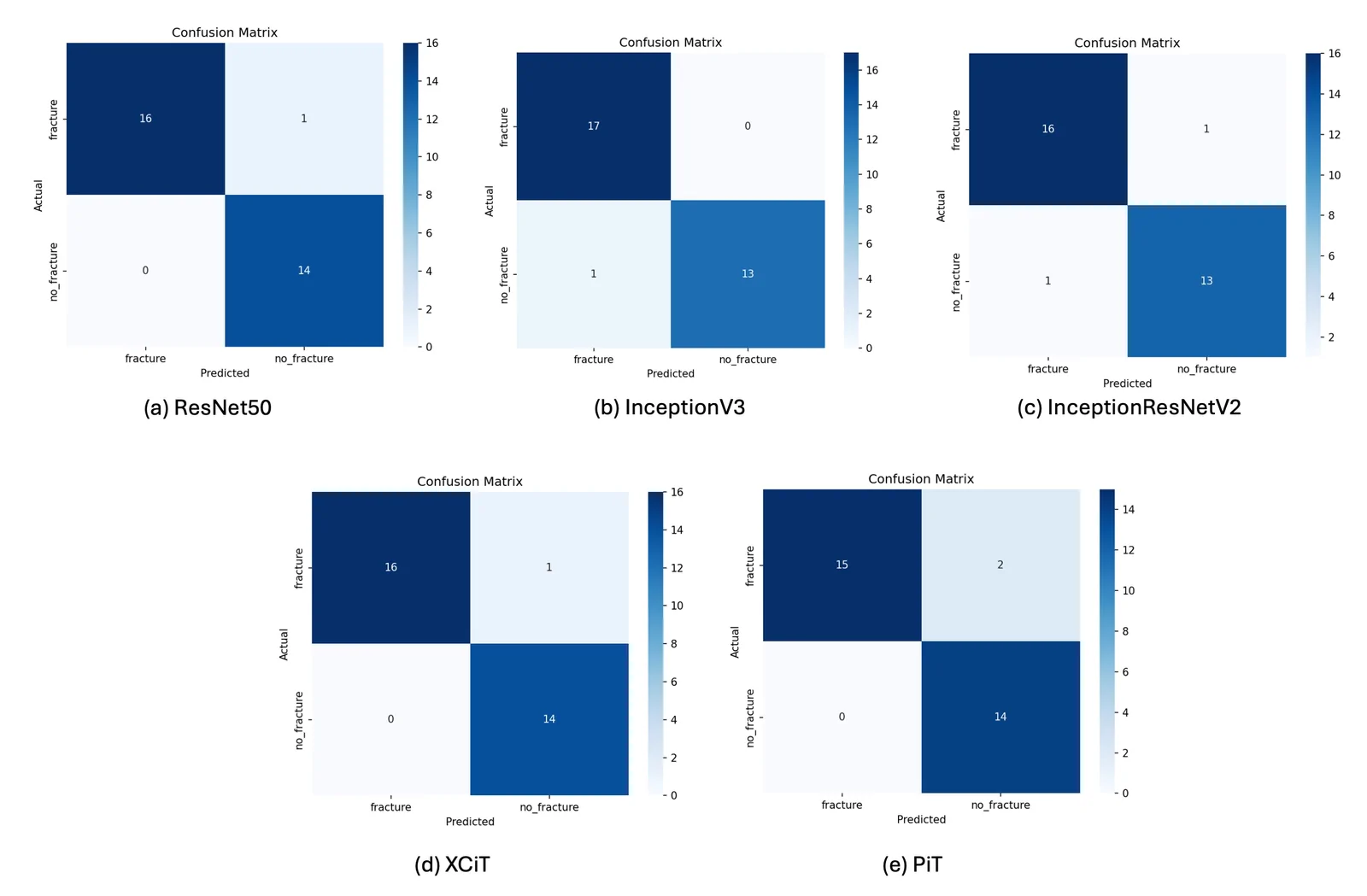
Comparison of confusion matrices for the five deep learning models in the AP view.

**Figure 3:**
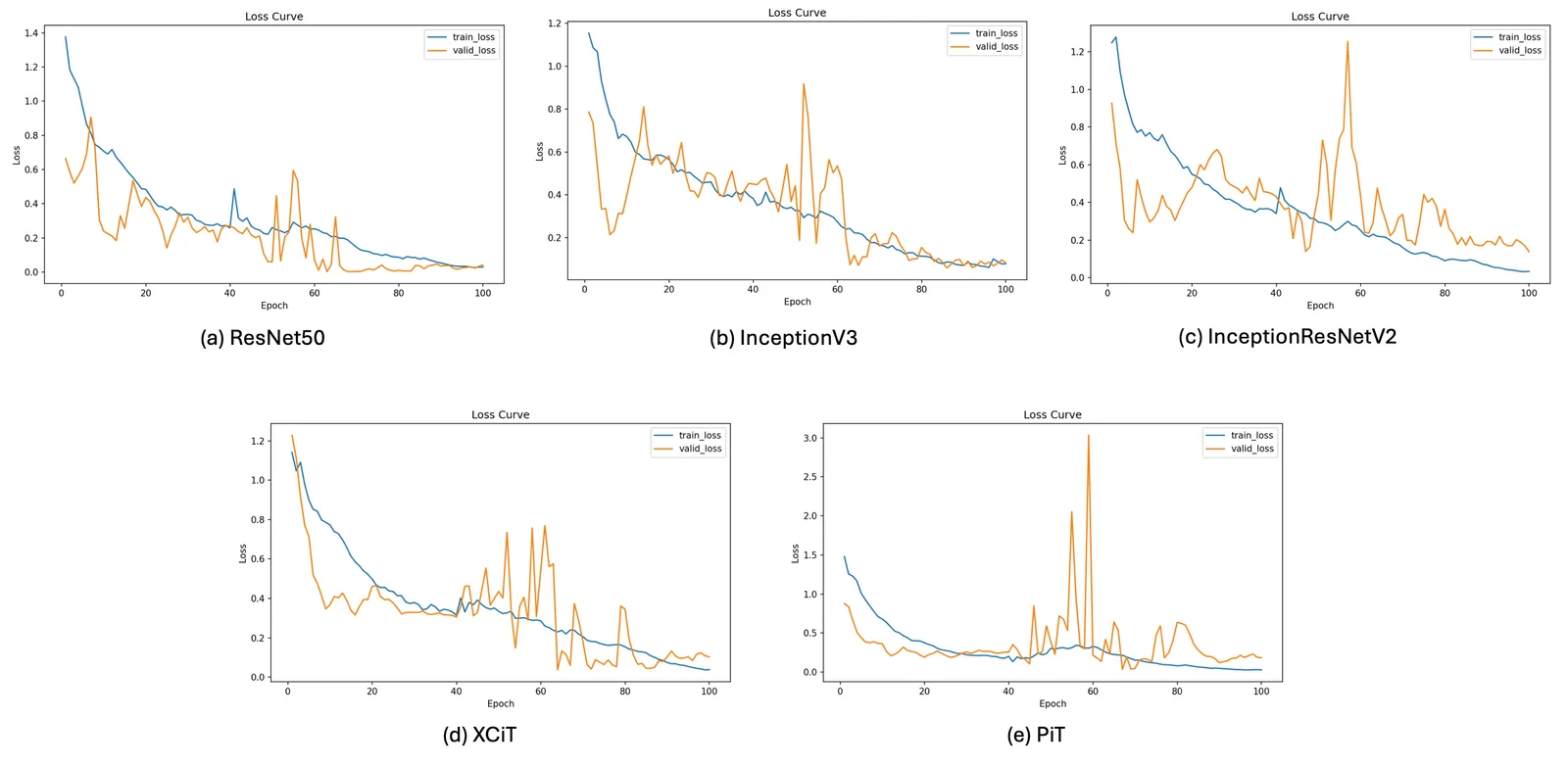
Training and validation loss curves for the five models in the AP view.

**Figure 4:**
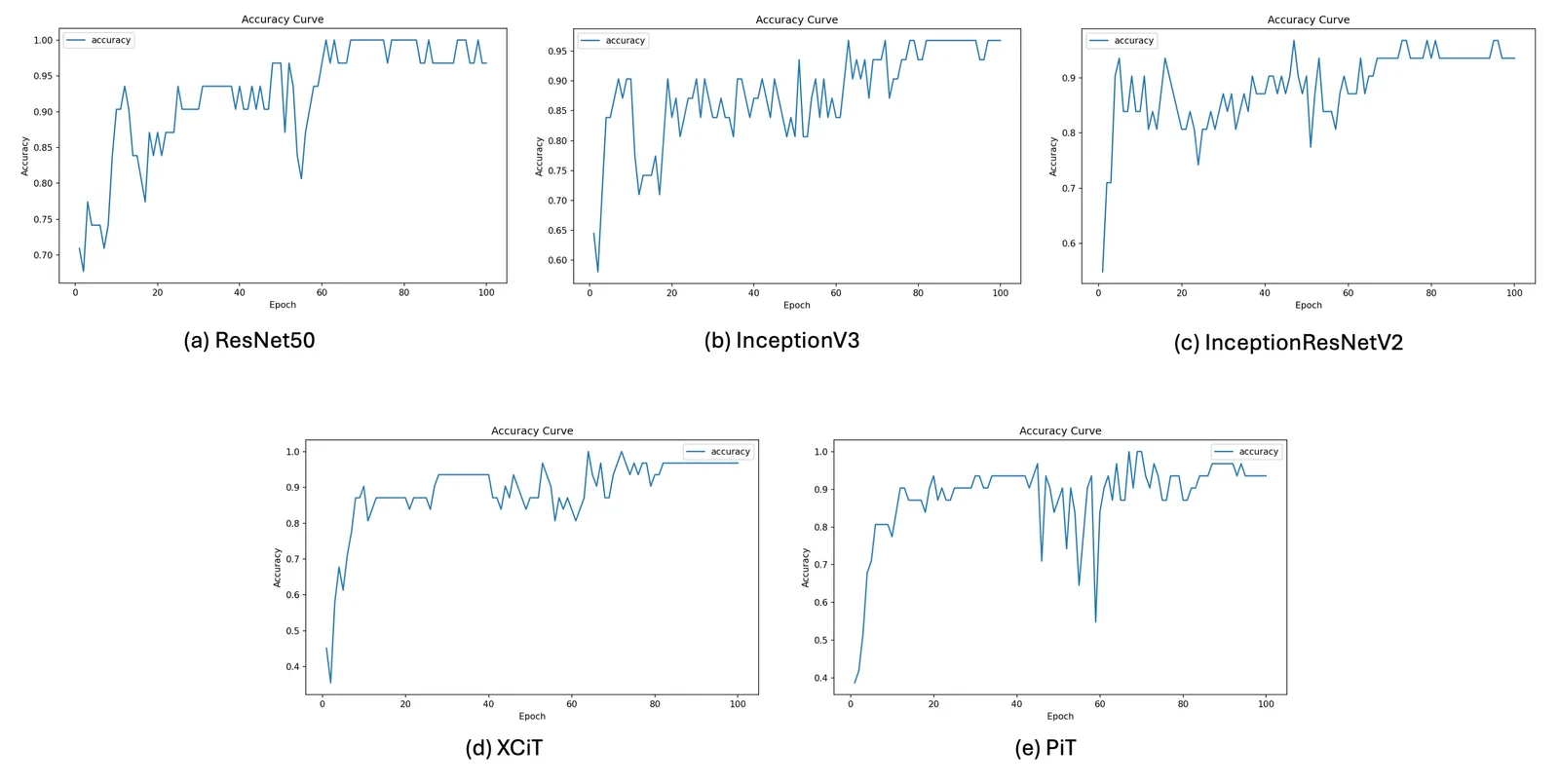
Validation accuracy curves for the five models in the AP view.

**Figure 5:**
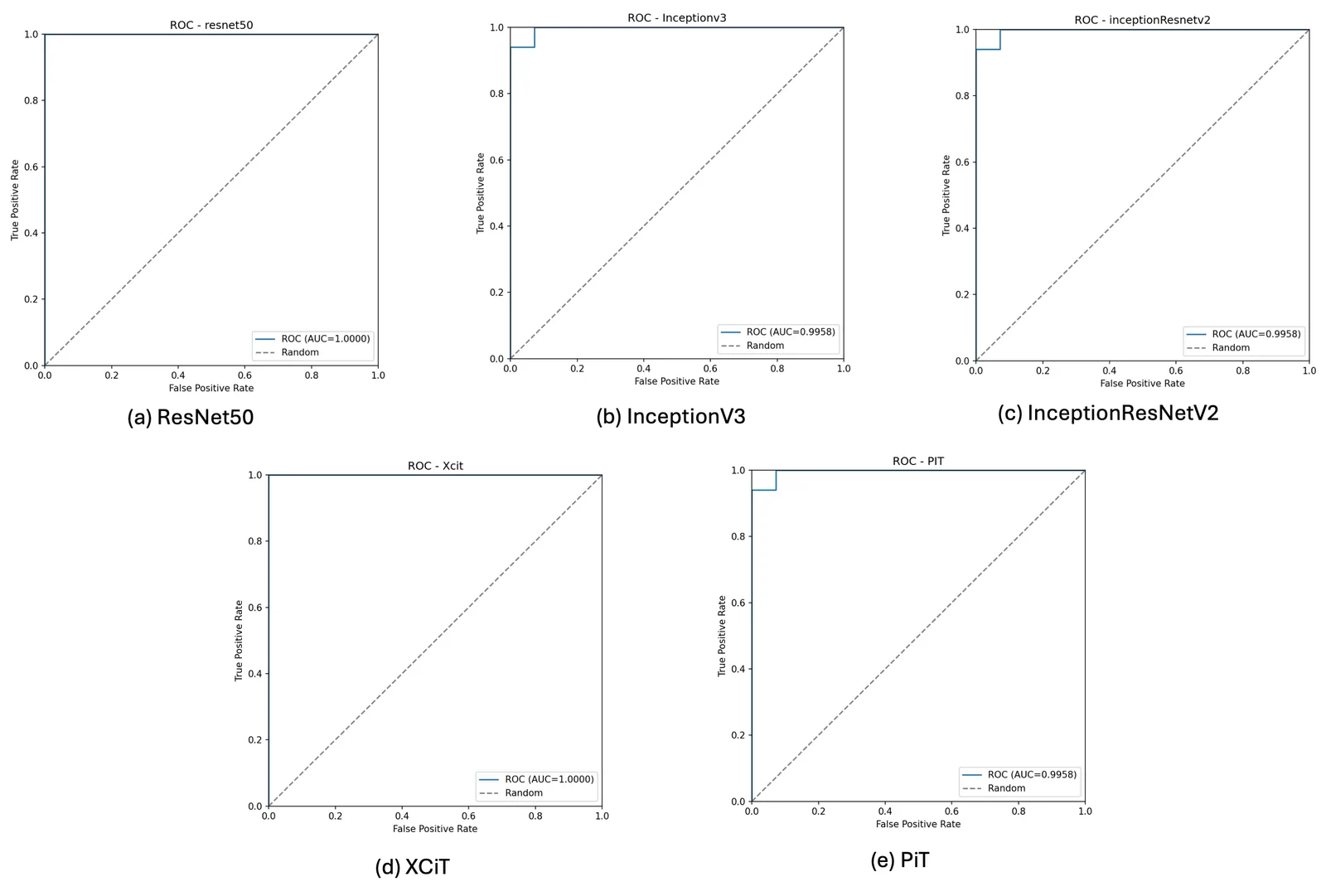
ROC curves for the five models in the AP view.

In the AP validation set, InceptionV3, ResNet50, and XCiT each achieved an accuracy of 96.8% (95% CI, 83.8–99.4%) As summarized in Table 1, InceptionV3 achieved 100.0% sensitivity (95% CI, 81.6–100.0%), identifying all 17 fracture images in this validation set, while its accuracy (96.8%) and F1-score (97.1%) were jointly highest and numerically highest, respectively, among the evaluated models. On the other hand, ResNet50 and XCiT exhibited 100.0% specificity and precision, with no false-positive predictions in the AP validation set, yielding an accuracy of 96.8%. ResNet50 and XCiT had identical values for all five classification metrics. In contrast, the PiT model showed lower sensitivity in this view (88.2%), with two false-negative predictions. AUC values were 1.0000 for ResNet50 and XCiT and 0.9958 for InceptionV3, InceptionResNetV2, and PiT. The confusion matrices, training and validation loss curves, validation accuracy curves, and ROC curves for the AP view are presented in Figure 2, Figure 3, Figure 4 and Figure 5, respectively.

### 4.2. Diagnostic Performance of Models in the Harris View

**Figure 6:**
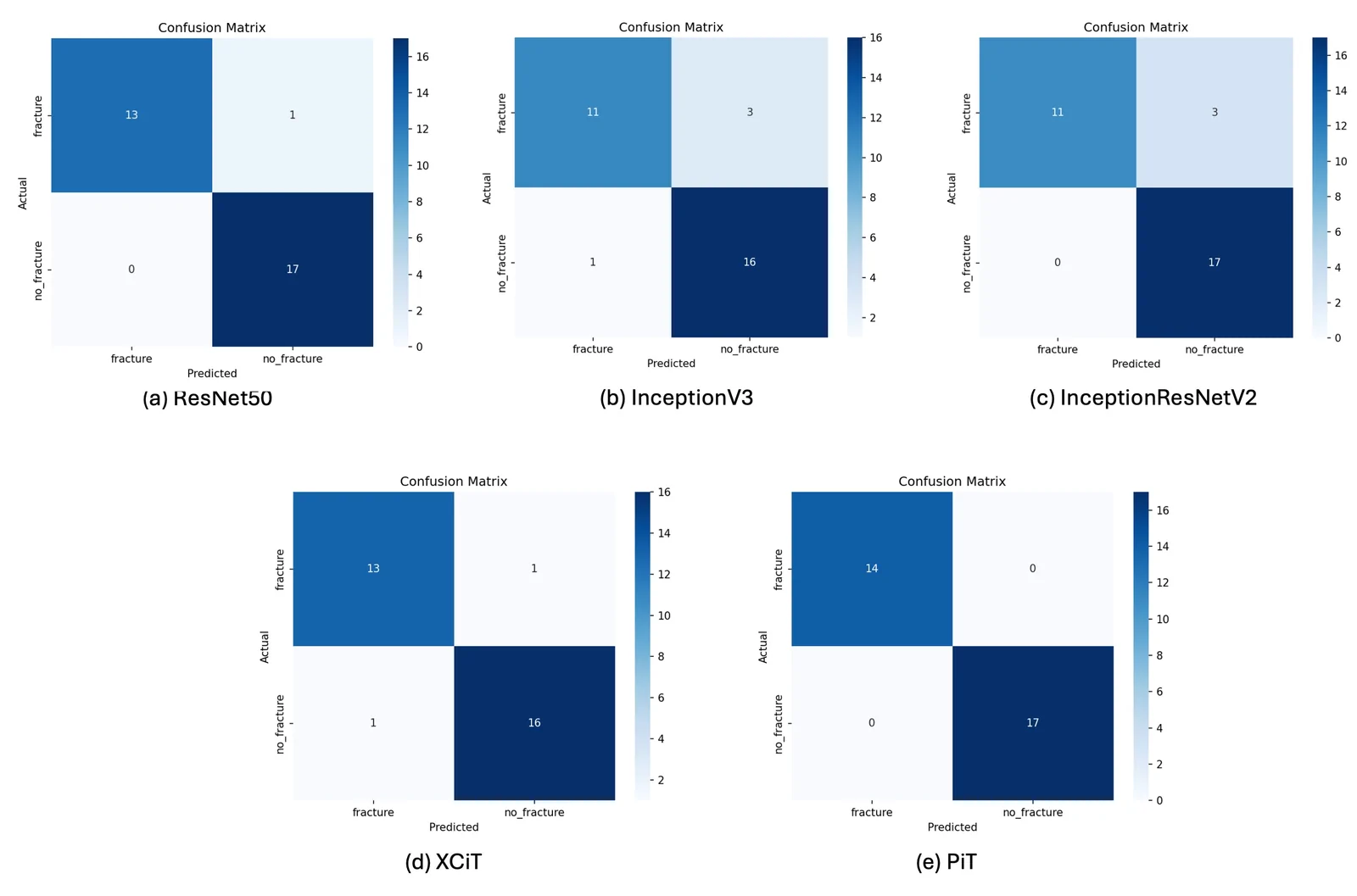
Confusion matrices for the five models in the Harris view.

**Figure 7:**
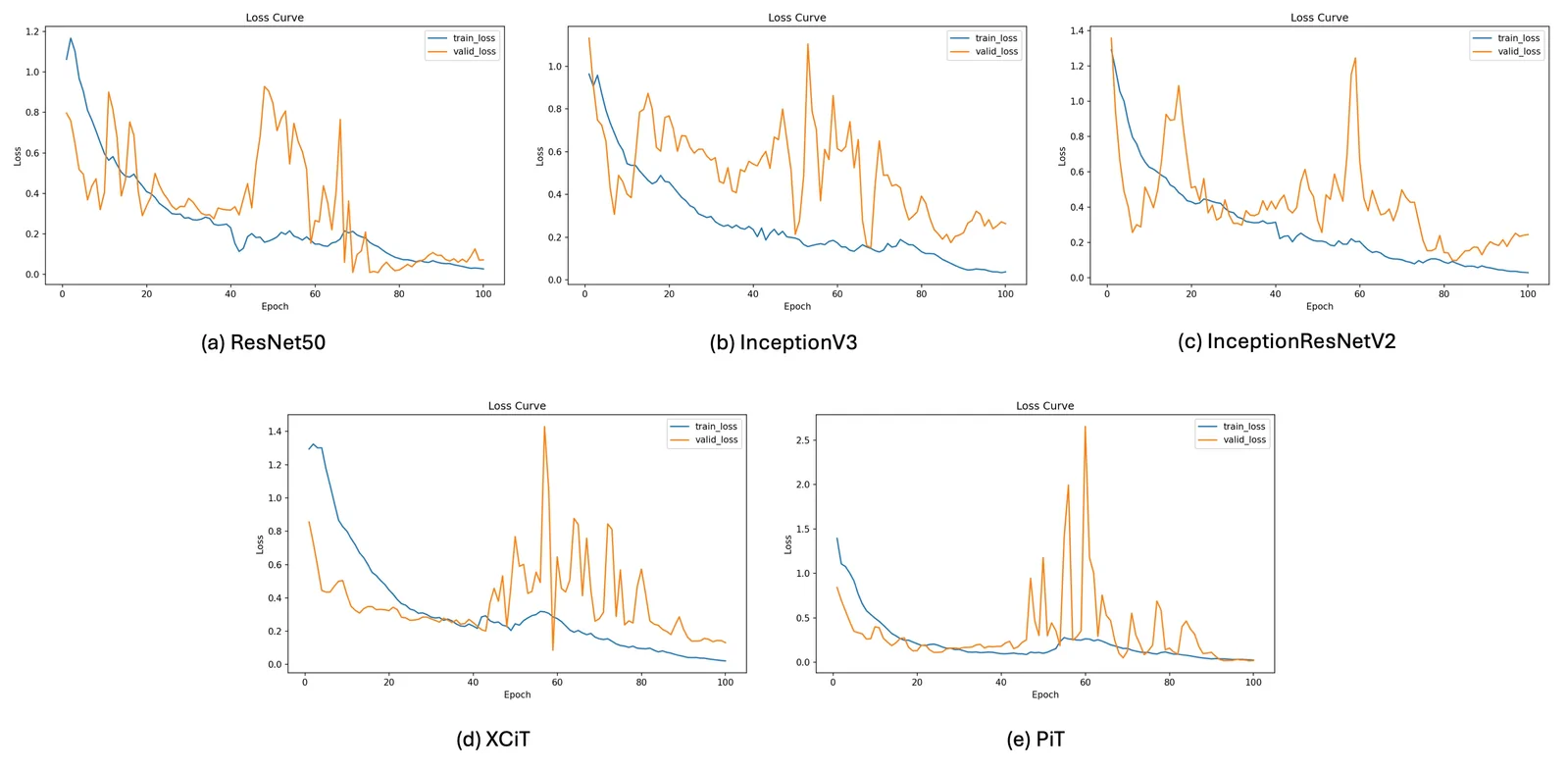
Training and validation loss curves for the five models in the Harris view.

**Figure 8:**
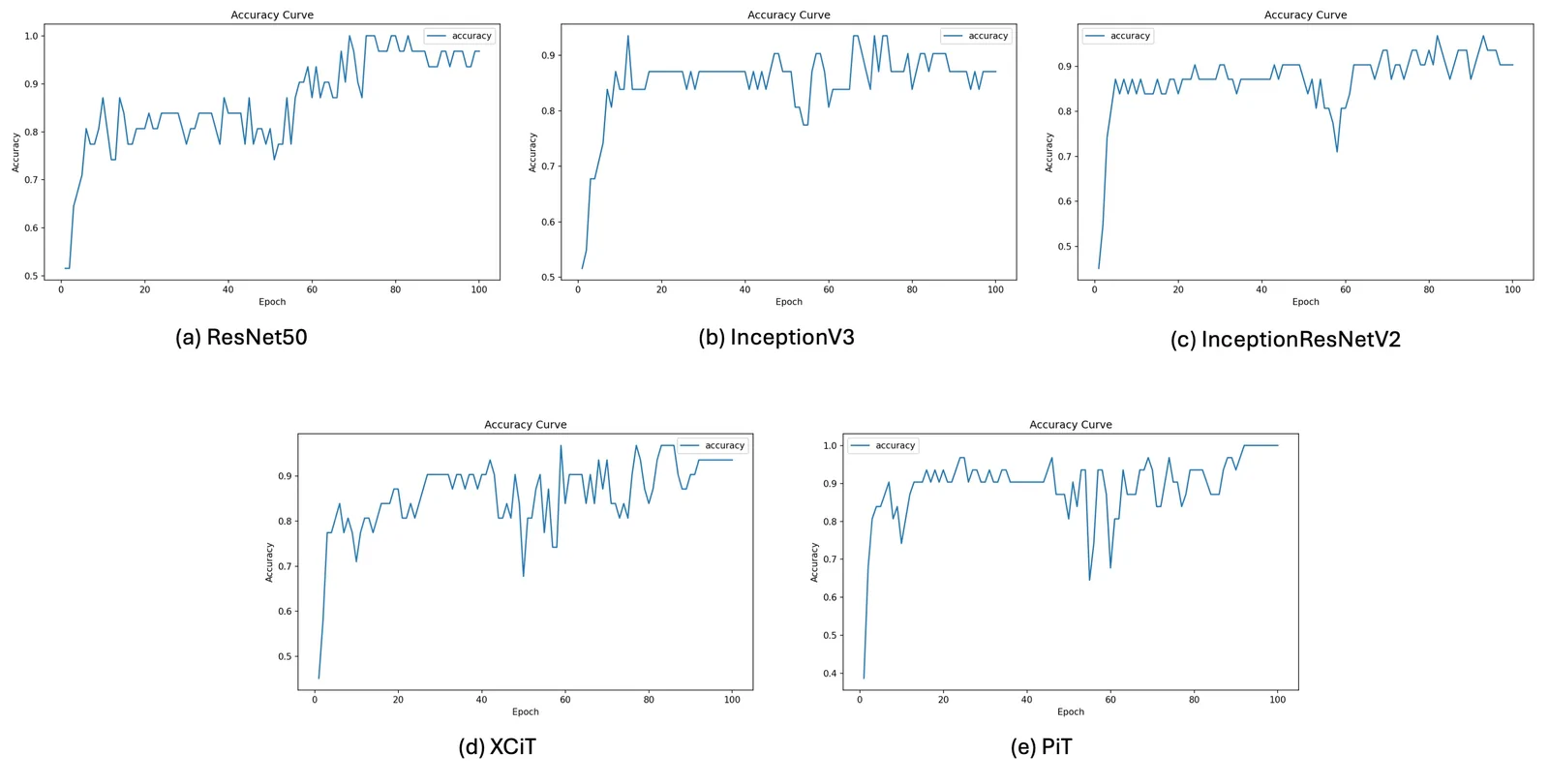
Validation accuracy curves for the five models in the Harris view.

**Figure 9:**
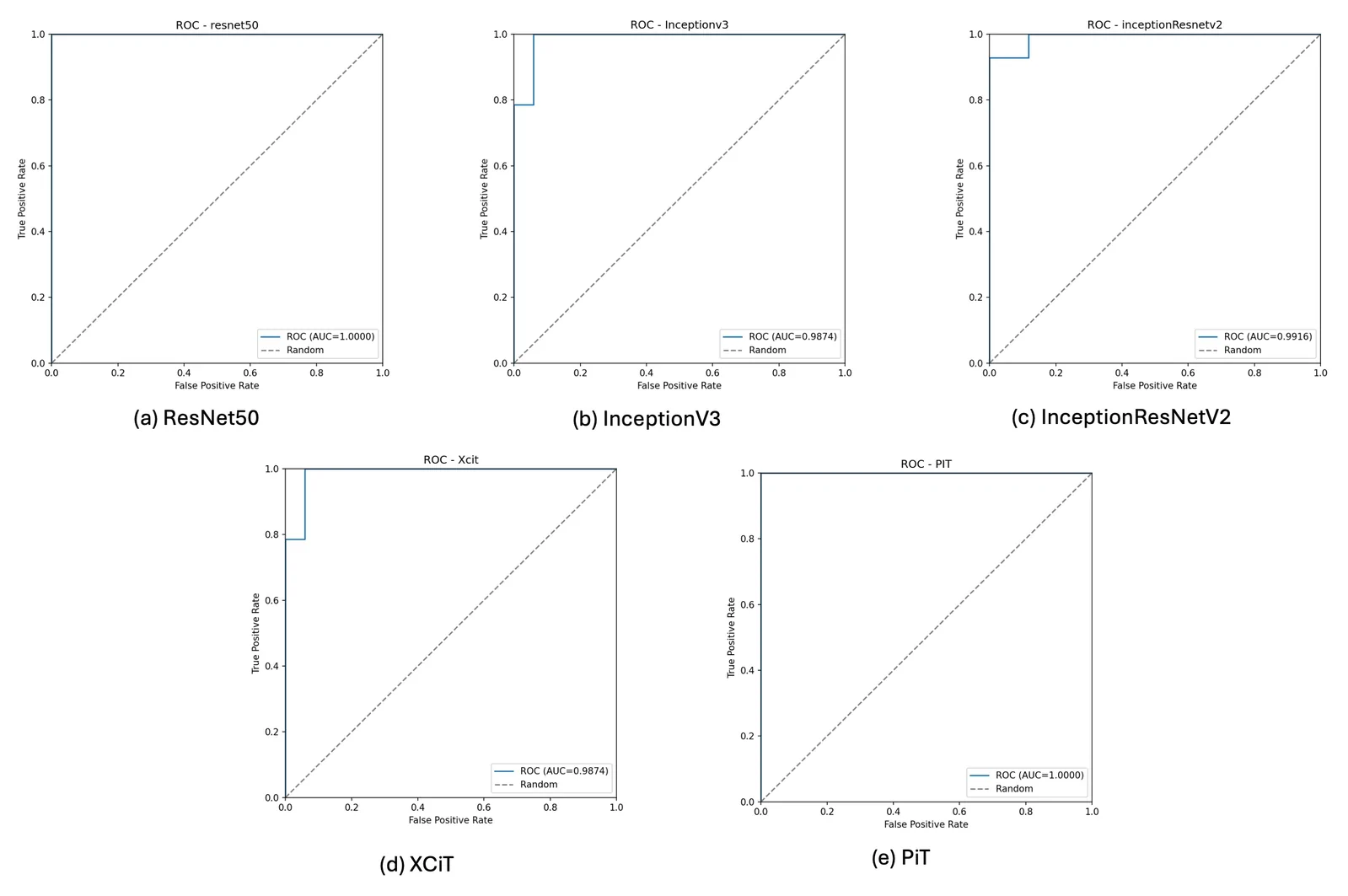
ROC curves for the five models in the Harris view.

In the Harris view, the Vision Transformer-based PiT model achieved the highest point estimates for the five classification metrics. PiT had sensitivity, specificity, precision, accuracy, and F1-score all reaching 100.0%, with no false-negative or false-positive predictions among the 31 validation images, as shown in Table 2. The 95% CI for its accuracy was 89.0–100.0%. The traditional CNN architecture, ResNet50, achieved a sensitivity of 92.9%, alongside specificity and precision of 100.0%, an accuracy of 96.8% and an F1-score of 96.3%. In comparison, InceptionResNetV2 and InceptionV3 demonstrated lower sensitivity (78.6% for both), with three false-negative predictions each. AUC values were 1.0000 for ResNet50 and PiT, 0.9916 for InceptionResNetV2, and 0.9874 for InceptionV3 and XCiT. The confusion matrices, training and validation loss curves, validation accuracy curves, and ROC curves for the Harris view are presented in Figure 6, Figure 7, Figure 8 and Figure 9, respectively.

### 4.3. Diagnostic Performance of Models in the Lateral View

**Figure 10:**
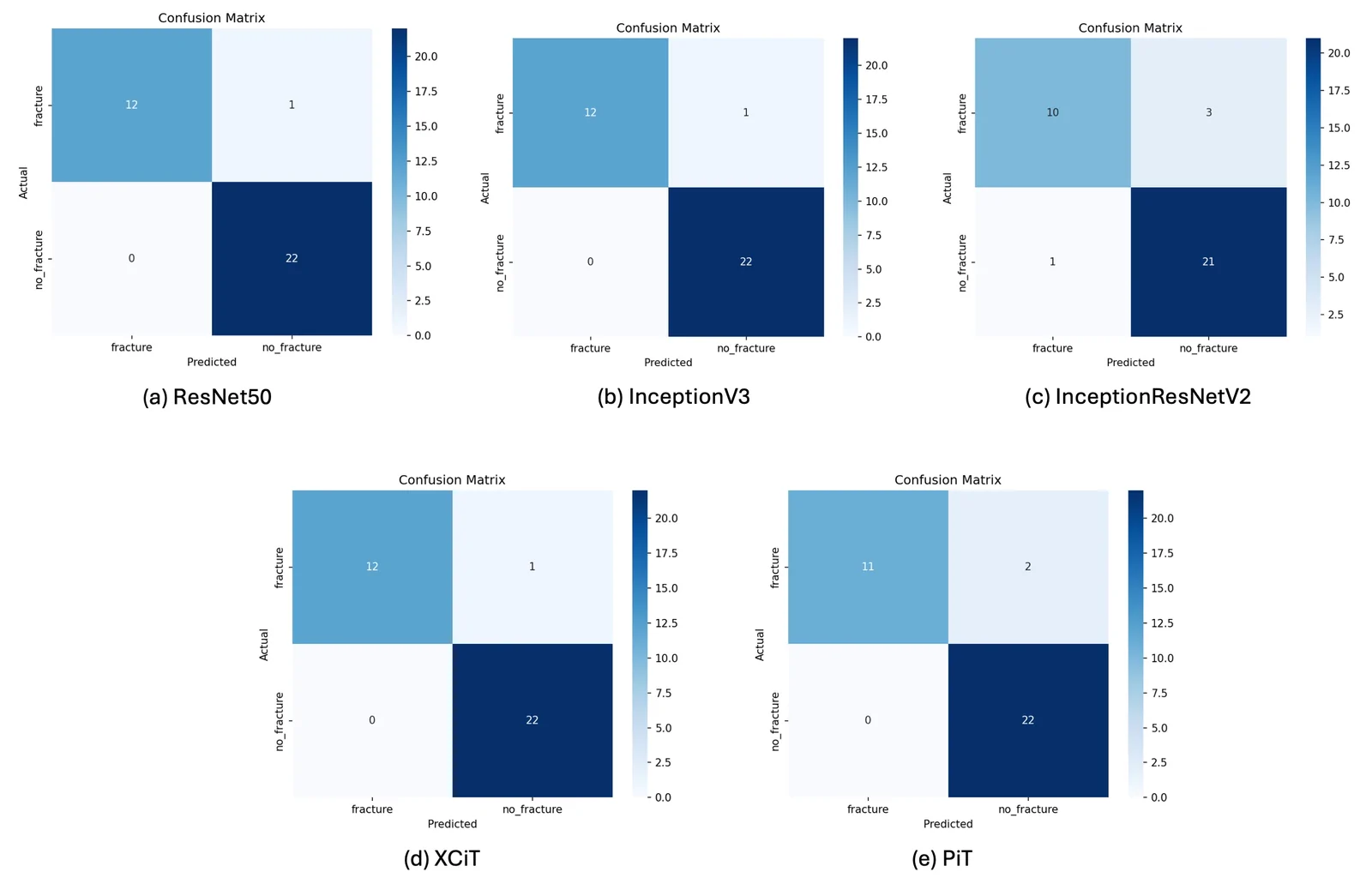
Confusion matrix for model performance in the lateral view.

**Figure 11:**
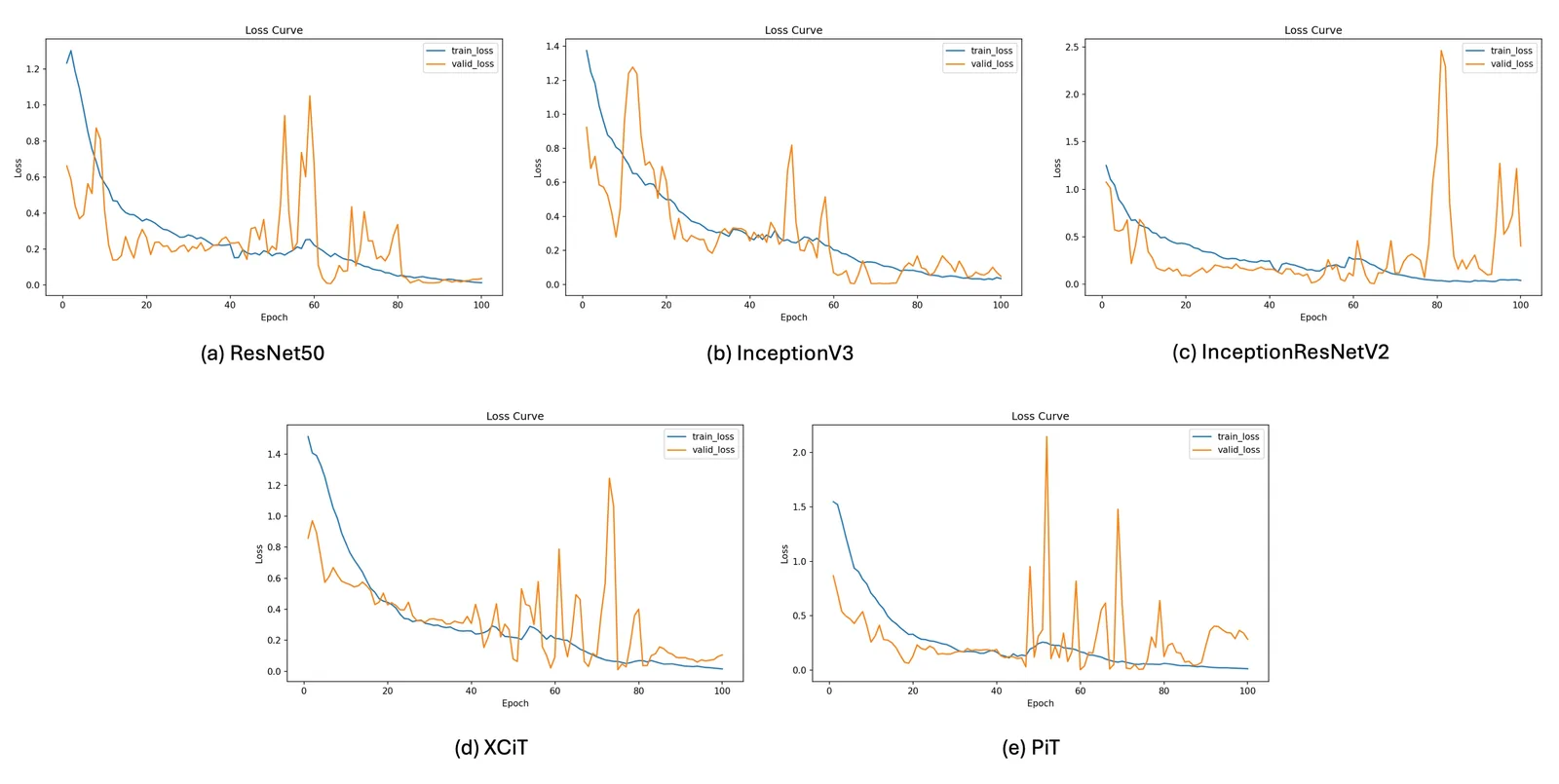
Training and validation loss curves for the five models in the lateral view.

**Figure 12:**
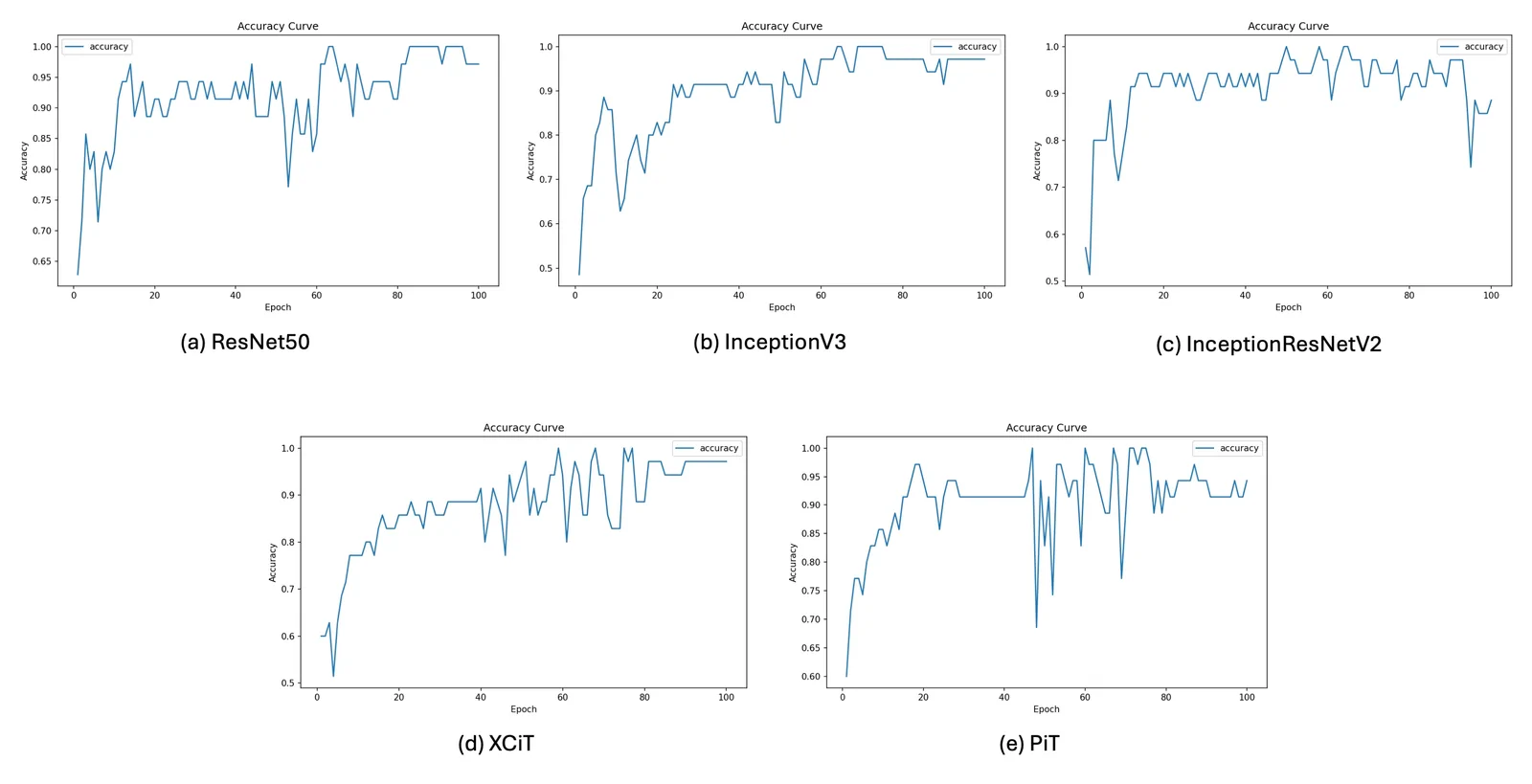
Validation accuracy curves for the five models in the lateral view.

**Figure 13:**
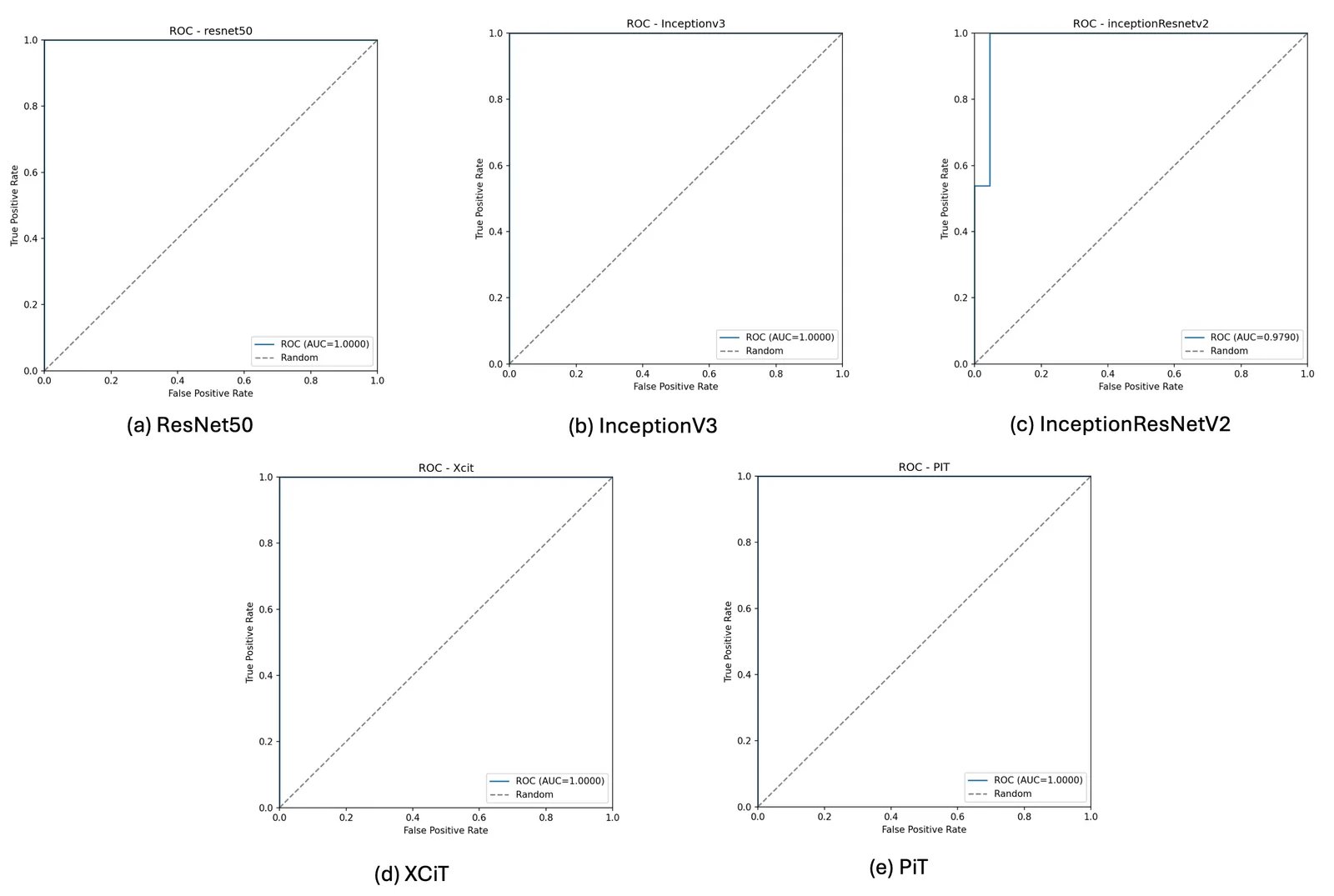
ROC curves for the five models in the lateral view.

For the Lateral view images, ResNet50, XCiT, and InceptionV3 demonstrated identical values for the five classification metrics, as detailed in Table 3. They all exhibited a sensitivity of 92.3%, specificity of 100.0%, precision of 100.0%, an accuracy of 97.1% and an F1-score of 96.0%. PiT achieved a sensitivity of 84.6%, an accuracy of 94.3%, and an F1-score of 91.7% in the Lateral validation set. Although InceptionResNetV2 maintained specificity of 95.5%, its sensitivity was 76.9%, the lowest sensitivity point estimate among the evaluated models in this view. AUC values were 1.0000 for ResNet50, InceptionV3, XCiT, and PiT and 0.9790 for InceptionResNetV2. The confusion matrices, training and validation loss curves, validation accuracy curves, and ROC curves for the Lateral view are depicted in Figure 10, Figure 11, Figure 12 and Figure 13, respectively.

### 4.4. Grad-CAM Visualization Analysis

To visualize the spatial distribution of model activation, this study employed Grad-CAM technology [15] to generate heatmaps. The red areas in the images represent higher Grad-CAM activation for the fracture class, while the blue areas denote lower activation.

**Figure 14:**
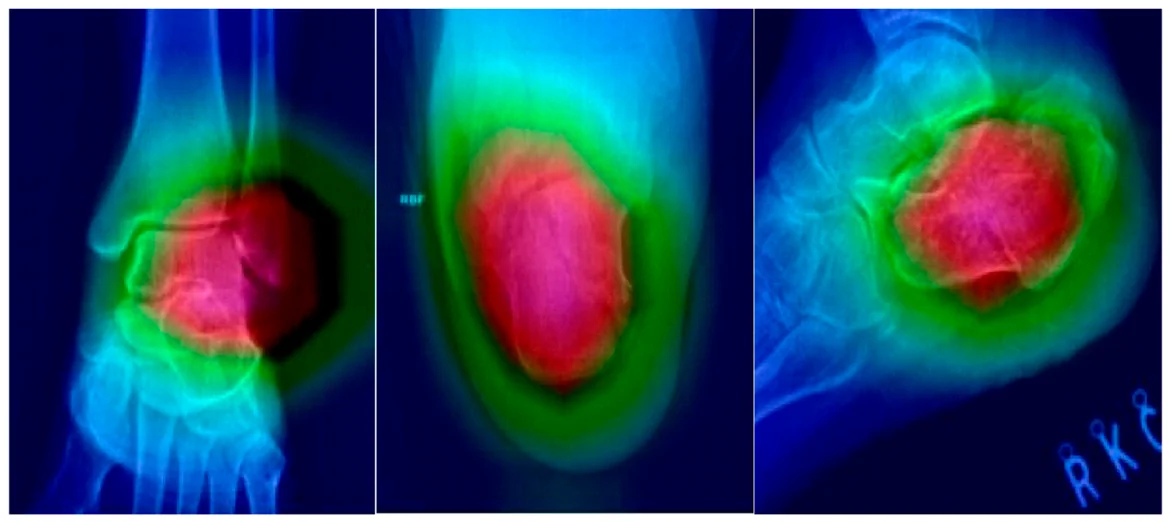
Grad-CAM visualization results for representative fracture cases in AP, Harris, and Lateral views. Warmer colors indicate higher Grad-CAM activation for the fracture class. In the illustrated examples, warmer-colored regions overlap the visible fracture sites. One representative true-positive image is shown per view.

In the representative true-positive cases, the heatmaps showed activation over the calcaneal region, with warmer-colored regions overlapping visible fracture sites and extending into surrounding portions of the calcaneal body. The activation patterns for the three radiographic views are illustrated in Figure 14 and described below:

AP View: In the representative AP image, warmer-colored regions overlapped the visible fracture site amid overlapping anatomical structures.

Harris View: In the representative Harris image, warmer-colored regions overlapped the visible fracture region, with activation extending across the calcaneus in the axial projection.

Lateral View: In the representative Lateral image, warmer-colored regions overlapped the visible fracture site and extended into adjacent portions of the calcaneal body.

## 5. Discussion

This study evaluated five deep learning models (ResNet50, InceptionV3, InceptionResNetV2, XCiT, and PiT) for diagnosing calcaneal fractures across three standard radiographic views. The results model specific performance patterns within the three validation sets. In the AP view, InceptionV3 achieved 100% sensitivity and the highest F1-score (97.1%), while ResNet50 and XCiT achieved 100% specificity. In the Harris view, PiT achieved 100% across five classification metrics, yielding the highest point estimates in this validation set. In the Lateral view, ResNet50, InceptionV3, and XCiT achieved identical classification metrics, with an accuracy of 97.1%. Overall, XCiT matched ResNet50 across the five classification metrics in the AP view and matched ResNet50 and InceptionV3 in the Lateral view, while PiT achieved favorable results in the Harris view. These findings provide a basis for further investigation of individual models across radiographic projections, rather than establishing a general distinction between CNN and Transformer performance.

The performance disparities observed in this study may partly relate to differences in the feature extraction mechanisms between CNNs and Vision Transformers. In the AP view and Lateral view, there is significant anatomical overlap between the calcaneus and surrounding tarsal bones (AP view), or a need to identify minute joint surface depressions and cortical margin fractures (Lateral view). The diagnostic focus relies on identifying “subtle fracture lines and high-frequency edge information” within complex backgrounds. The inductive bias of CNNs may be useful for extracting these local features. InceptionV3 utilizes its multi-scale convolutional kernels to simultaneously capture image features of various sizes, which provides a possible hypothesis for its performance in the AP view [18]. ResNet50 utilizes residual connections to facilitate feature learning in deep networks [8]; however, the present analysis did not directly establish which features accounted for its observed performance. These architectural explanations should therefore be regarded as hypotheses rather than demonstrated mechanisms.

In contrast, the Harris (axial) view emphasizes overall calcaneal geometry, including widening and varus/valgus deformities, making global spatial relationships relevant alongside local edge features. Vision Transformers can model spatial relationships through their self-attention mechanisms. In particular, PiT combines attention-based feature modeling and hierarchical pooling [21]. This provides a possible hypothesis for its 100% classification scores in the Harris view, compared with the 78.6% sensitivity of InceptionV3. Overall, the findings are more consistent with a favorable result for PiT in one validation set than with a general advantage of Transformers for the Harris view. The matching results of XCiT and the leading CNN models in the AP and Lateral views further argue against a simple CNN-versus-Transformer distinction.

Although AI application in orthopedic imaging is increasingly popular, research specifically targeting the radiographic diagnosis of calcaneal fractures remains relatively sparse. Previous literature has investigated fractures of the wrist [10], ankle [12], or hip [11], as well as calcaneal fracture diagnosis using X-ray images [13]. In the present study, the best-performing models achieved accuracies at least 96.8% across all three separately evaluated views (AP, Harris, Lateral), including 100% accuracy in the Harris view. These findings support further investigation of automated calcaneal fracture detection. Direct comparisons with previous studies require caution because patient selection, imaging datasets, and evaluation procedures differ.

Compared with previous AI studies on calcaneal fractures, this study provides two key contributions. First, building on previous X-ray-based research [13], we evaluate CNNs and Vision Transformers (XCiT and PiT) under shared principal training conditions, using the same validation images for all five models within each view Second, this study reports performance separately for AP, Lateral, and Harris radiographs. For example, PiT achieved 100% accuracy in the Harris view but 84.6% sensitivity in the Lateral view, whereas InceptionV3 achieved 100% sensitivity in the AP view but 78.6% in the Harris view. These observations motivate further investigation of model performance across projections. No feature-level fusion, decision-level fusion, or ensemble was evaluated in this study. Integrating the three views is a direction for future research, and any benefit should be assessed using patient-matched images and an independent test set.

The findings of this study have potential clinical implications. The missed diagnosis rate of calcaneal fractures during initial interpretation can reach 10% [4], highlighting the potential value of AI-assisted screening. Models with high sensitivity, such as InceptionV3 in the AP view and PiT in the Harris view, are candidates for further evaluation as diagnostic aids, while the high specificity of ResNet50 supports further assessment of its ability to distinguish fracture from non-fracture images. Moreover, Grad-CAM visualizations showed warmer-colored regions overlapping visible fracture sites in the representative examples. These visualizations provide qualitative information about the spatial distribution of model activation. By providing classification outputs and accompanying activation maps, these models may contribute to the development of AI-assisted fracture assessment tools, subject to further clinical evaluation.

The main contributions of this work are summarized as follows: 1. Classification performance within each radiographic view under shared principal training conditions. 2. CNN and Transformer comparison: Five CNN- and Transformer-based models are compared to investigate their classification performance within each radiographic view under shared principal training conditions. 3. Explainable AI analysis: Grad-CAM is employed to visualize model activation in representative fracture images, including warmer-colored regions overlapping visible fracture sites.

Several limitations should be acknowledged. This retrospective, single-center study included a small, deliberately balanced cohort, with only displaced fractures and radiograph-based controls, potentially introducing spectrum bias and limiting generalizability. Demographic data, fracture classifications, and counts of CT-confirmed fractures were unavailable. Model evaluation relied on a single training–validation split per view, without an independent test set, repeated experiments, or external validation. Small, unmatched validation cohorts, the absence of paired statistical testing, and potential augmentation-related data leakage further limit interpretation. Image preprocessing may also affect clinical applicability. Grad-CAM assessment was qualitative, without systematic quantitative validation, and only binary classification was evaluated without multi-view fusion. Future studies should incorporate representative trauma cohorts, documented patient-level partitioning, and independent multicenter validation.

## 6. Conclusions

This study demonstrates the potential of deep learning for detecting displaced calcaneal fractures through separate evaluations of AP, Harris, and Lateral radiographs. XCiT matched the classification performance of leading CNN models in the AP and Lateral validation sets, while PiT achieved the highest classification scores in the Harris validation set. These model-specific findings provide a foundation for further development of AI-assisted calcaneal fracture detection. Grad-CAM visualizations showed activation overlapping visible fracture sites in representative images. Future research should investigate multi-view integration and evaluate these models in larger, independent multicenter cohorts.

## Figures and Tables

**Table 1:** Comparison of diagnostic performance among deep learning models in the AP view.

Model	Sensitivity	Specificity	Precision	Accuracy	F1 Score	AUC
ResNet50	94.1 [73.0, 99.0]	100.0 [78.5, 100.0]	100.0 [80.6, 100.0]	96.8 [83.8, 99.4]	97.0 [84.4, 99.5]	1.0000
InceptionV3	100.0 [81.6, 100.0]	92.9 [68.5, 98.7]	94.4 [74.2, 99.0]	96.8 [83.8, 99.4]	97.1 [85.2, 99.5]	0.9958
InceptionResNetV2	94.1 [73.0, 99.0]	92.9 [68.5, 98.7]	94.1 [73.0, 99.0]	93.5 [79.3, 98.2]	94.1 [80.4, 98.4]	0.9958
XCiT	94.1 [73.0, 99.0]	100.0 [78.5, 100.0]	100.0 [80.6, 100.0]	96.8 [83.8, 99.4]	97.0 [84.4, 99.5]	1.0000
PiT	88.2 [65.7, 96.7]	100.0 [78.5, 100.0]	100.0 [79.6, 100.0]	93.5 [79.3, 98.2]	93.8 [79.3, 98.3]	0.9958

**Table 2:** Comparison of diagnostic performance among deep learning models in Harris view.

Model	Sensitivity	Specificity	Precision	Accuracy	F1 Score	AUC
ResNet50	92.9 [68.5, 98.7]	100.0 [81.6, 100.0]	100.0 [77.2, 100.0]	96.8 [83.8, 99.4]	96.3 [81.3, 99.4]	1.0000
InceptionV3	78.6 [52.4, 92.4]	94.1 [73.0, 99.0]	91.7 [64.6, 98.5]	87.1 [71.1, 94.9]	84.6 [64.9, 94.2]	0.9874
InceptionResNetV2	78.6 [52.4, 92.4]	100.0 [81.6, 100.0]	100.0 [74.1, 100.0]	90.3 [75.1, 96.7]	88.0 [68.8, 96.1]	0.9916
XCiT	92.9 [68.5, 98.7]	94.1 [73.0, 99.0]	92.9 [68.5, 98.7]	93.5 [79.3, 98.2]	92.9 [76.6, 98.1]	0.9874
PiT	100.0 [78.5, 100.0]	100.0 [81.6, 100.0]	100.0 [78.5, 100.0]	100.0 [89.0, 100.0]	100.0 [87.9, 100.0]	1.0000

**Table 3:** Comparison of diagnostic performance among deep learning models in Lateral view.

Model	Sensitivity	Specificity	Precision	Accuracy	F1 Score	AUC
ResNet50	92.3 [66.7, 98.6]	100.0 [85.1, 100.0]	100.0 [75.8, 100.0]	97.1 [85.5, 99.5]	96.0 [80.0, 99.3]	1.0000
InceptionV3	92.3 [66.7, 98.6]	100.0 [85.1, 100.0]	100.0 [75.8, 100.0]	97.1 [85.5, 99.5]	96.0 [80.0, 99.3]	1.0000
InceptionResNetV2	76.9 [49.7, 91.8]	95.5 [78.2, 99.2]	90.9 [62.3, 98.4]	88.6 [74.0, 95.5]	83.3 [62.4, 93.8]	0.9790
XCiT	92.3 [66.7, 98.6]	100.0 [85.1, 100.0]	100.0 [75.8, 100.0]	97.1 [85.5, 99.5]	96.0 [80.0, 99.3]	1.0000
PiT	84.6 [57.8, 95.7]	100.0 [85.1, 100.0]	100.0 [74.1, 100.0]	94.3 [81.4, 98.4]	91.7 [73.2, 97.8]	1.0000

## Data Availability

The data that support the findings of this study are available from the corresponding author upon reasonable request.
